# Metacognition as a mediator of the relation between family SES and language and mathematical abilities in preschoolers

**DOI:** 10.1038/s41598-024-60972-0

**Published:** 2024-05-06

**Authors:** Mélanie Maximino-Pinheiro, Iris Menu, Esther Boissin, Lys-Andréa Brunet, Carlo Barone, Grégoire Borst

**Affiliations:** 1 Laboratory for the Psychology of Child Development and Education (LaPsyDE) – CNRS: UMR8240, University Paris Cité, Paris, France; 2 Laboratory for Interdisciplinary Evaluation of Public Policies (LIEPP), Sciences Po, Paris, France; 3 Centre for Research on Social Inequalities (CRIS) – CNRS: UMR7049, Sciences Po, Paris, France; 4 French University Institute (Institut Universitaire de France), Paris, France

**Keywords:** Metacognition, SES, Preschoolers, Academic achievement, Language, Mathematics, Psychology, Human behaviour

## Abstract

The effect of family socioeconomic status (SES) on academic achievement in literacy and numeracy has been extensively studied with educational inequalities already witnessed in preschoolers. This is presumably explained by the effect of family SES on cognitive and socioemotional abilities associated with academic achievement. Metacognition which refers to knowledge and regulation skills involving reflexivity about one's own cognitive processes is one of these abilities. However, most of the studies investigating the association between metacognition and academic achievement have focused on school-aged students and studies with younger students are only emerging. Meanwhile, the association between family SES and metacognition abilities has surprisingly received little attention regardless of participants’ age. The aim of this study was to explore the associations between family SES, metacognition, language and mathematical abilities in preschoolers aged 5 to 6. We provide the first evidence that the effect of family SES on preschoolers’ language and mathematical abilities is mediated by the effect of family SES on their metacognitive abilities. The implications for future research, education and policies aiming at reducing educational inequalities are discussed.

## Introduction

The effect of family socioeconomic status (SES) on academic achievement in literacy and numeracy has been extensively studied^1–4^ with educational inequalities already witnessed in preschoolers^5–8^. These inequalities are attributed to multiple factors stratified by socioeconomic background, family structure, economic and cultural resources as well as family interactions and parental practices^9–12^. This research shows that family SES affects academic achievement because of its effect on the cognitive and socioemotional abilities which are associated with academic success. For instance, researchers have paid increasing attention to the link between family SES and executive functions (including inhibitory control, cognitive flexibility and working memory), self-control, perseverance or interpersonal abilities^5,6,13,14^. However, the effect of family SES on children and adolescents’ metacognitive abilities has received little attention despite the fact that they are also critical for academic achievement^15^.

Metacognition refers to knowledge and regulation skills involving reflexivity about one's own cognitive processes^16–18^. Metacognitive knowledge includes knowledge about people, tasks and strategies. The first refers to knowledge that individuals have about people as learners (e.g., knowing their own strengths and weaknesses as a learner) and the general properties of cognition (e.g., knowing that memorization is based on many repetitions of the information). The second and the third ones cover knowledge about the tasks and the strategies characteristics (e.g., knowing that some tasks require more cognitive resources than others, knowing that some strategies are more relevant to achieve a given task). Metacognition skills include planning, monitoring and control, and evaluation. Planning refers to setting the goal of the activity and selecting the most appropriate strategies to achieve it. Monitoring and control occur as the activity is being carried out and allows one to determine if everything is going as planned and to adjust if necessary. Finally, evaluation allows one to assess what has been learned, the effectiveness of the procedure used, and to determine how to improve in the future.

Meta-analyses indicate that metacognition is correlated with academic achievement in literacy and numeracy^19,20^. Importantly, up to date only a few studies on metacognition in educational settings have focused on preschoolers and young school-aged children^21,22^. This is explained in part because authors proposed originally that metacognitive abilities emerged from the age of 8 and continue to develop during adolescence^23^. This conception of a late emergence and development of metacognitive abilities was in part due to the difficulty to assess them in young children^24^. Lately, by adopting new measures (i.e., an observation grid of children’s behaviors and a checklist to fill in by teachers), Whitebread and colleagues provided evidence for metacognitive abilities as early as 3 years of age^25,26^.

Meanwhile, the few studies that investigated the relation between metacognition and family SES report a positive association between the two^15^. For instance, in students from the 4^th^ to the 8^th^ grade, metacognitive knowledge, regulation of cognition and the use of metacognitive strategies are positively associated with facets of SES such as parental education^27,28^. Data from PISA 2009 (Programme for International Student Assessment) on 15-year-olds also reveals that students from high-SES countries tend to use more metacognitive strategies, and the ones which are most strongly related to academic achievement, than students from low-SES countries^29^. In young school-aged children, one study reported that family SES was strongly related to 2nd graders metacognitive abilities^30^. Finally, to our knowledge, the only study conducted with preschoolers aged 4 to 5 revealed lower metacognitive awareness and expression of thinking in children from low-SES families^31^.

While the effect of family SES on school achievement and on metacognition has been documented, as well as the one between school achievement and metacognition, no study to date investigated whether the effect of the family SES of preschoolers on their language and mathematical abilities—two foundational abilities of future school achievement^32–34^—could be partly mediated by the effect of family SES on their metacognitive abilities.

To do so, we recruited 90 French preschoolers aged 5 to 6 and we measured family SES (parent’s educational level and occupational status as reported by the parents), metacognition (metacognitive knowledge and skills measured respectively through an interview conducted by the experimenters and a questionnaire completed by the teachers), language (vocabulary, phonology and grammar) and mathematical (counting, numeration and arithmetical operations) abilities both assessed by the experimenters with standardized cognitive tests. We computed composite scores for each of the four domains through principal component analyses (for two studies using a similar approach^35,36^). We then used structural equation modeling (SEM) to run a double mediation analysis^37^ with metacognition as the common mediator of the relation between family SES and language abilities, and between family SES and mathematical abilities.

In line with previous studies, we expected that the family SES would have an impact on preschoolers’ metacognition and that their metacognitive abilities would also have an effect on their language and mathematical abilities. Finally, we hypothesized that preschoolers’ metacognition would constitute a mediator of the relation between family SES and their language and mathematical abilities.

## Results

To compute the composite score for each of the four domains (SES, metacognition, language and mathematical abilities), we used a principal component analysis (PCA) approach. These index scores were then predicted by extracting the first principal component (PC1). Table 1 summarizes the eigenvalues of the PC1 for each domain. The PC1 explains a large majority of the variance for SES, metacognition and language (≥ 70%) and a majority of the variance for mathematics (58%). Table 2 provides the bivariate correlations and the descriptive statistics for all variables.

**Table 1:** Eigenvalues of the PC1 for each composite score computed by PCA.

Index	Eigenvalue	Proportion of variance explained	Factor loading	
SES	1.74	0.87	Parent’s educational level	0.93
			Parent’s occupational status	0.93
Metacognition	1.49	0.74	Metacognitive skills	0.86
			Metacognitive knowledge	0.86
Language	2.09	0.70	Vocabulary	0.82
			Phonology	0.82
			Grammar	0.86
Mathematics	1.74	0.58	Counting	0.80
			Numeration	0.67
			Operation	0.80

**Table 2:** Bivariate correlations and descriptive analyses for all variables.

	1	2	3	4	5	6	7	8	9	10	11	12	13	14
1. Index of SES	_													
2. Parent’s educational level	0.93	_												
3. Parent’s occupational status	0.93	0.74	_											
4. Index of Metacognition	0.42	0.43	0.36	_										
5. Metacognitive skills	0.42	0.42	0.36	0.86	_									
6. Metacognitive knowledge	0.31	0.31	0.26	0.86	0.48	_								
7. Index of Language	0.61	0.63	0.52	0.68	0.66	0.51	_							
8. Vocabulary	0.49	0.49	0.41	0.62	0.57	0.49	0.82	_						
9. Phonology	0.45	0.42	0.41	0.65	0.63	0.5	0.82	0.49	_					
10. Grammar	0.59	0.63	0.46	0.44	0.45	0.3	0.86	0.57	0.56	_				
11. Index of Mathematics	0.41	0.36	0.4	0.64	0.66	0.44	0.62	0.39	0.64	0.52	_			
12. Counting	0.46	0.38	0.47	0.6	0.65	0. 38	0.56	0.31	0.61	0.49	0.8	_		
13. Numeration	0.14	0.12	0.14	0.32	0.29	0.23	0.24	0.15	0.31	0.14	0.68	0.31	_	
14. Operation	0.3	0.3	0.28	0.54	0.54	0.38	0.59	0.42	0.51	0.53	0.8	0.48	0.32	_
*N*	85	85	85	89	90	89	89	89	90	90	85	85	85	85
*M*	0	14.29	53.13	0	62.91	12.21	0	19.12	17.76	18.13	0	4.54	9.49	7.26
*SD*	1	3.14	21.79	1	15.19	4.29	1	4.6	4.61	5.58	1	2.36	3.22	3.25
*Min*	− 3.39	0	14.64	− 2.23	29	3	− 3.56	4	1	1	− 2.65	0	1	1
*Max*	1.26	17	85.41	2.15	88	22	1.53	29	23	27	2.31	8	14	15

For the correlations, coefficients ≥ 0.23 are significant at level 0.05, coefficients ≥ 0.29 are significant at level 0.01 and coefficients ≥ 0.36 are significant at level 0.001. Index scores are computed through principal component analyses including standardized variables resulting in a mean of 0 and a standard deviation of 1.

### Mediation analysis

**Figure 1:**
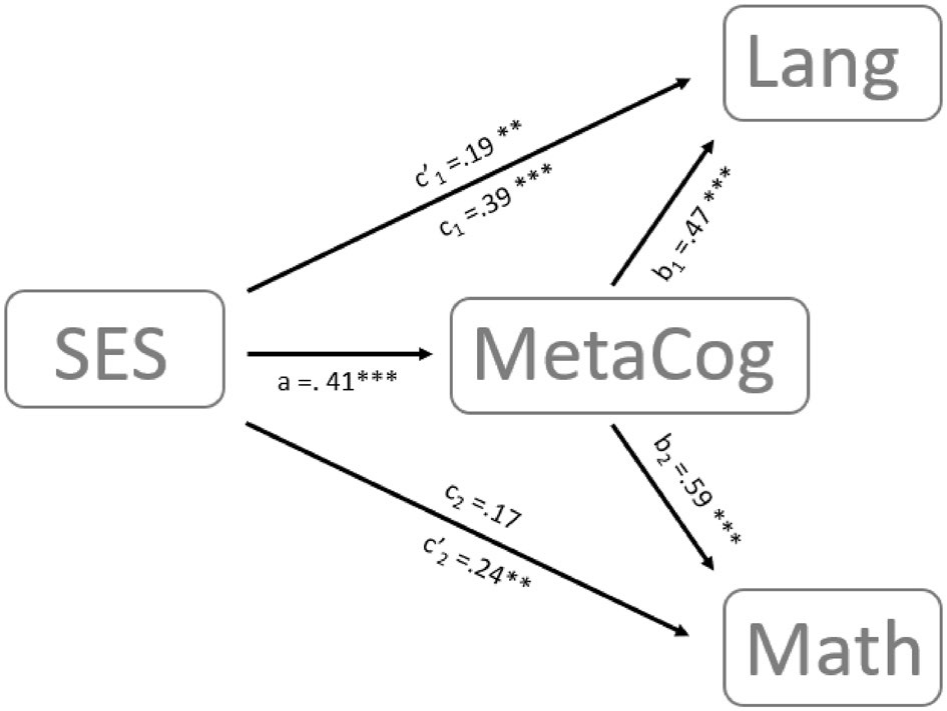
Double mediation analysis of the relations between SES and language, and SES and math abilities with metacognition as mediator. Paths a, b_1_, b_2_, c_1_ and c_2_ report the beta weight for each corresponding direct effect. Paths c'_1_ and c'_2_ report the beta weight for each corresponding indirect effect. Significance levels: **p < 0.01 and ***p < 0.001.

The analysis revealed an adequate fit of the model: χ^2^(6) = 134.92 (*p* < 0.001), RMSEA = 0, SRMR = 0 and CFI = 1. The results of the double mediation are presented in Fig. 1. We found a significant effect of SES on children's metacognition (path a, *β* = 0.41 (*SE* = 0.09), *p* < 0.001) and a significant effect of metacognition on both language (path b_1_, *β* = 0.47 (*SE* = 0.10),* p* < 0.001) and mathematical abilities (path b_2_, *β* = 0.59 (*SE* = 0.11), *p* < 0.001). The direct effect of SES on children's language abilities was significant (path c_1_, *β* = 0.39 (*SE* = 0.09), *p* < 0.001) as the indirect one via metacognition (path c'_1_, *β* = 0.19 (*SE* = 0.06), *p* < 0.01), revealing that the effect of family SES on language abilities is partially mediated by the effect of SES on metacognition. For mathematical abilities, the indirect effect of SES through metacognition was significant (path c'_2_, *β* = 0.24 (*SE* = 0.08), *p* < 0.01) but not the direct one (path c_2_, *β* = 0.17 (*SE* = 0.11), *p* = 0.12), suggesting that the effect of family SES on mathematical abilities is fully mediated by the effect of SES on metacognition.

## Discussion

The aim of the present study was to explore the associations between family SES, metacognition, language and mathematical abilities in preschoolers aged 5 to 6. As expected, we found that family SES had an effect on preschoolers’ metacognition as reported in 4 to 5 years of age children^31^ and in school-aged children^27–30^. Moreover, we found that metacognitive abilities were related to language and mathematical abilities in line with previous studies reporting a relation between metacognitive abilities and academic outcomes in preschoolers^21,22^, and older students from primary school to college^19,20^. Note that while language abilities are not academic outcome per se they are foundational abilities for literacy^33,38^.

Importantly, we provide the first evidence that the effect of family SES on preschoolers’ language and mathematical abilities is mediated by the effect of family SES on their metacognitive abilities. Further studies are needed to replicate these findings among a larger sample—with particular attention given to the fit of the model which may be influenced by the relatively small sample size in this study—but also to determine whether metacognitive abilities mediate the relation between family SES and academic outcomes in older age groups. Indeed, the associations between metacognition and academic achievement^19,20^, and metacognition with SES^28^, tend to vary with age being stronger in young children than older ones.

Further studies are also needed to determine the mechanisms by which family SES affects children's metacognition. Parenting practices might be one of such mechanisms. Indeed, studies have provided evidence that parenting practices can promote autonomy to experiment on their own, to engage in cognitively stimulating activities but also to develop metacognitive abilities by using metacognitive questions^39^. Importantly, high-SES parents appear to ask more metacognitive questions to their young children by encouraging them to plan, think of relevant strategies to use, self-monitor and self-reflect (e.g., *How are you going to figure out the next piece? Isn’t it smarter to start from the bottom and go up? What do you think the problem is there?*)^40^ which could explain in turn why children from high-SES families develop better metacognitive abilities.

The mediation of metacognitive abilities of the association between family SES and preschoolers’ language abilities need to be interpreted with caution because language abilities can also be considered as prerequisites for the development of metacognition and in particular for the development of metacognitive knowledge^41–43^ which are encoded, stored and restituted verbally. Since metacognitive skills refer to the procedural aspects of metacognition, we can assume that they rely less on language abilities than metacognitive knowledge. However, in our study, metacognitive skills were assessed by teachers and they might have been biased by children's language abilities, rating as more metacognitively skilled a child who also more expresses verbally the strategies that s/he uses. To address this limitation, further studies need to include measures of metacognitive skills which rely less on verbal abilities. Finally, future studies should also control for potential confound which might be at the root of the mediation observed in the present study such as parental language abilities or parental cognitive abilities that might in turn affect both parental SES and children’s metacognitive abilities.

Taken together, our findings suggest that metacognition could be a promising lever to reduce educational inequalities from the earliest age. Consistent with this assumption, interventions in school-aged children promoting their metacognition and self-regulated learning—which is a broader framework frequently associated with metacognition including metacognitive as well as motivational and cognitive aspects^44–46^—have been shown to benefit their academic performance^47–49^. Implementing such interventions to reduce educational inequalities is supported by studies suggesting that students from low SES background tend to benefit more from the interventions but only after a delay^47^.

To date, only a few recent studies have tried to improve metacognition and self-regulated learning in preschoolers^50–52^. They provided encouraging results on the promotion of metacognitive abilities with different approaches targeting children in class, teachers’ training and/or parents. However, further studies are needed to explore potential transfers to academic performance and potentially differentiated effects according to children’s family SES in this specific age group. Since the effect of SES on metacognitive abilities seems to vary with age, being stronger in children than adolescents^28^, it might be even more interesting to explore this issue in young children.

In conclusion, our study provides the first evidence that the effect of family SES on preschoolers’ language and mathematical abilities is mediated by the effect of family SES on their metacognitive abilities. This result has important implications for educators and policymakers suggesting that metacognition could constitute a promising lever to improve academic achievement^53^ as well as to reduce educational inequalities from the earliest age.

## Method

### Participants

Ninety French preschoolers were recruited in public preschools (46 girls, *Mean* age = 5.5 ± 0.3 years). Children’s parents provided a written informed consent to participate to this study which was approved by the ethical committee (CER, Université Paris Cité). The study was carried out in accordance with national and international norms that govern the use of human research participants.

### Materials and procedure

The questionnaires used to measure family SES and children's metacognitive skills were completed by parents and teachers respectively. The interview assessing metacognitive knowledge and the tests assessing children's language and mathematical abilities were administered in schools by trained research assistants.

#### Socioeconomic status

A SES composite score was computed using a method similar to the one used by the OCDE for the PISA surveys^36,54^ by selecting the highest level of education and the highest occupational status between both parents. First, each mother and father’s levels of education were converted into years of education ranging from 0 (no degree) to 17 (Master's degree and more). Second, mother and father's occupations were coded according to the International Standard Classification of Occupations (ISCO-08)^55^ using the 3-digit-level. ISCO-08 codes were then matched with their corresponding values in the International Socio-Economic Index of occupational status (ISEI-08)^56,57^ using the R software package *occupar*^58^. ISEI-08 is a classification ranking occupations based on the average level of education and earnings of workers (e.g., 11 for subsistence crop farmers, 51 for sports and fitness workers, 89 for medical doctors). For homemakers, students and unemployed people, an ISEI-08 value of 17 corresponding to the category of ‘elementary workers’ was attributed, consistent with the OECD method.

#### Metacognition

We focused on children’s metacognitive knowledge and metacognitive skills. Metacognitive knowledge was measured using the *Metacognitive Knowledge Interview* (McKI)^59^. Children first performed a construction game with three progressive levels of complexity and had then to answer questions assessing their metacognitive knowledge about people (e.g., *Would these puzzles be hard for another kid your age? Why/why not?*), tasks (e.g., *Would the puzzle be easier with bigger or smaller pieces? Why?*) and strategies (e.g., *If I think about how the pieces would fit together before I try, will the puzzle be easier? Why/why not?*). Children's responses were audio-recorded and rated following the original scoring codebook as 0 = not at all metacognitive, 1 = partially metacognitive and 2 = appropriately metacognitive. This study was part of a broader project including 176 interviews administered with the same sample at two different times. Thirty percent of these 176 interviews were double coded by two trained raters. Considering their high level of agreement (intraclass correlation = 0.97), each rater coded half of the rest of the sample. Metacognitive skills were assessed through the *Children’s Independent Learning Development* checklist for children aged 3–5 (CHILD 3–5)^25^ filled in by the teachers. They had to rate the frequency of each child's behaviors like planning (e.g., *Plans own tasks, targets and goals*), monitoring and control (e.g., *Monitors progress and seeks help appropriately*) and evaluation (e.g., *Can speak about how they have done something or what they have learnt*) as 1 = never, 2 = sometimes, 3 = usually or 4 = always. The McKI and the CHILD 3–5 are two measures of metacognition with good reliability (α = 0.76 and α = 0.97 respectively) and external validity (i.e., associations reported with other measures of metacognition, theory of mind and executive functions). We also checked the reliability of these measures on our own data and we found good reliability for both the McKI (α = 0.70) and the CHILD 3–5 (α = 0.97).

#### Language abilities

Language abilities in vocabulary, phonology and grammar were assessed using tests from the standardized French battery Health Check—Evaluation of the Development for Schooling between 5 and 6 (BSEDS 5–6, *Bilan de Santé—Evaluation du Développement pour la Scolarité de 5 à 6 ans*)^60,61^. In the ‘vocabulary’ test, the experimenter said a word and the child had to designate the most appropriate picture. In the ‘phonological awareness’ test, children had to find the rhyming word with the one given by the experimenter (e.g., *Which word rhymes with ‘balloon’ between ’basket’, ‘afternoon’ and ‘school’?*), to count and to remove syllables from different words (e.g., *Repeat ‘paper’ without saying [pa]*). Then, grammar abilities were assessed with the ‘morpho-syntactic production’ test in which the experimenter started a sentence describing a picture, and the child has to complete the end by using the appropriate pronouns, space–time markers, gender and number, verb conjugation and types of sentences. The BSEDS 5–6 was previously validated and showed good reliability (inter-rater reliability leading to *r* > 0.85 for the sub-tests used in our study) and good sensitivity, specificity and predictive values to detect children with and without language difficulties. The reliability of the measures on our own data was good for the phonological awareness (α = 0.87) and grammar sub-tests (α = 0.80). The reliability was only marginally acceptable for the vocabulary subtest (α = 0.62). We note however that the descriptive results in our sample are similar to the one reported in the original battery (*Mean* ± *SD* of the battery = 19 ± 4,5 ; *Mean* ± *SD* in our study = 19,12 ± 4,6).

#### Mathematical abilities

For mathematics, we used tasks assessing counting, numeration and arithmetical operations abilities from the standardized French version of the *Diagnostic Test of Basic Skills in Mathematics* (TEDI-MATH, *Test diagnostique des compétences de base en mathématiques*)^62^. We measured counting abilities by administering the tasks ‘counting as far as possible’, ‘counting forward to an upper bound’ (e.g., count to 6), ‘counting forward from a lower bound’ (e.g., count from 3) and ‘counting forward from a lower bound to an upper bound’ (e.g., count from 5 to 9). Numeration abilities were then assessed through the two subsections ‘writing Arab numbers under dictation’ and ‘reading aloud Arab numbers’ from the ‘transcoding’ task. Finally, children's acquisitions in arithmetical operations were rated with the task ‘operations presented on pictures’ (e.g., 6 flowers—4 flowers) and the subsection ‘basic additions’ from the task ‘operations presented in arithmetical format’ (e.g., 2 + 2). The TEDI-MATH was previously validated and showed good reliability (α = between 0.70 and 0.99 for the sub-tests used in our study) and validity (i.e., associations between the sub-tests and the children's level of mathematics assessed by their teacher). The reliability of the measures on our own data was good for the counting (α = 0.76), numeration (α = 0.86) and arithmetical operations sub-tests (α = 0.80).

### Data analysis

Data analyses were conducted in the R software^63^ using the following packages: *tidyverse*^64^, *psych*^65^, *Hmisc*^66^ and *lavaan*^67^.

#### Index score computation

We created an index score for each construct of interest by running a principal component analysis (PCA) including the standardized collected variables^35,36^. For the SES index, we included the highest parental level of education and occupational status. For the index of metacognition, we ran the PCA with the scores of metacognitive knowledge and metacognitive skills. For the index of language abilities, we included the scores in vocabulary, phonology and grammar. For the index of mathematical skills, we included the scores in counting, numeration and arithmetical operations. These index scores were then predicted by extracting the first principal component (PC1). The relevance of this method was confirmed by checking the proportion of variance explained by the PC1 and the correlations between the collected variables and the PC1.

#### Mediation analysis

Using structural equation modeling (SEM), we conducted a double mediation analysis^37^ with metacognition as the common mediator of the relation between family SES and language abilities, and between family SES and mathematical abilities. The model was estimated applying the Robust Maximum Likelihood estimation (MLR). To deal with missing values, we used the Full Information Maximum Likelihood approach (FIML). The fit of the model was then checked with the χ2 test, the Root Mean Square Error of Approximation (RMSEA), the Standardized Root Mean Residual (SRMR) and the Comparative Fit Index (CFI). The fit is considered good when χ2 test is non-significant, RMSEA and SRMR < 0.05 and CFI > 0.95^68^.

## Data Availability

The datasets generated and analyzed for the present study are available from the corresponding author.
